# Plastically Deformed Tubes Subjected to Mechanical Expansion Processes

**DOI:** 10.3390/ma17112524

**Published:** 2024-05-24

**Authors:** Zijian Zhao, Abdel-Hakim Bouzid, Nor Eddine Laghzale

**Affiliations:** 1 Ecole de Technologie Superieure, 1100 Notre-Dame Ouest, Montreal, QC H3C 1K3, Canada; hakim.bouzid@etsmtl.ca; 2 L’École Nationale Supérieure d’Arts et Métiers Rabat, Mohammed V University in Rabat, Rabat 10106, Morocco; n.laghzale@um5r.ac.ma

**Keywords:** tube expansion, finned tubes, deformation energy, beam on elastic foundation, elasto-plastic material behavior

## Abstract

In engineering, the stress state of expanded tubes is crucial for ensuring structural integrity and preventing stress corrosion cracking. The analysis of stresses and strains in tubes subjected to mechanical expansion using an ogive bullet is essential, yet existing theoretical methods for estimating the stress distributions, especially with spherical and ogive shapes, are sparse. This study explores the expansion of 3/8 inch copper and stainless-steel tubes using an expanding bullet, where tangential and longitudinal strains are measured. A novel analytical approach is introduced to evaluate the stresses and strains, segmenting the tube into three zones, each analyzed with a distinct theory. Validation is achieved through an axisymmetric finite element model that employs a multi-linear kinematic hardening material behavior. The analytical model also estimates the expanding mandrel’s push force, which is then compared with the results from numerical simulations and experimental data, showing good agreement across methods.

## 1. Introduction

Tube forming, a mechanical process involving plastic deformation, encompasses expansion, reduction, bending, die forming, and hydroforming. These work-hardening processes, essential for generating residual stresses, are widely used in the production of joint assemblies in heat exchangers, steam generators, boilers, and other pressure vessels. The manufacturing of tube-to-tubesheet and tube-to-fin assemblies, where joint stiffness is achieved through plastic deformation, often relies on empirical trial and error [1].

The tube expansion forming process, particularly crucial in heat exchanger production and assembly of tube-to-tubesheet and tube-to-fin parts, utilizes mechanical rolling, hydraulics, and expansion with mandrels. These methods are vital in the fabrication of tubular heat exchangers and gas coolers. However, if not properly controlled, tube expansion can result in high residual stresses and the potential for micro-cracks. Studies on the elasto-plastic behavior and estimation of residual stresses in tube-to-fins assemblies are limited, especially those involving the analytical modeling of the tube’s various expanded zones during the mechanical expansion process with mandrels.

In the literature, the stresses and deformations of the expanded tub-to-fin assembly are analyzed using experimental and numerical methods, predominantly using the finite element method [2,3,4,5]. This process, characterized by elasto-plastic material behavior with different strain hardening exponents, involves expanding the tube to close the gap between the tube and fin hole, ensuring proper adhesion for increased joint stiffness, structural integrity, and heat transfer [6]. Although tube expansion forming is an age-old process, research in this field is limited. Sawczuck and Hodge’s comparative study in 1960 on yield conditions for circular cylindrical shells under axisymmetric radial pressure initiated a series of investigations [7]. The expansion process impacts four zones: residual stress, contact, transition, and unexpanded zones [8]. These zones, especially those undergoing work hardening and experiencing stresses beyond yield, warrant closer examination.

Bouzid et al. proposed a methodology for estimating stresses in hydraulically expanded tube-to-tubesheet joints, accounting for similar zones produced by the expansion process and modeling the joint loading and unloading phases separately [9]. Departing from traditional theoretical methods, Liu et al. developed a new theoretical model for tube expansion based on energy dissipation theory [10]. With recent applications requiring more durable materials like stainless steel for CO_2_ cooled exchangers, there is a growing need for research on the mechanical expansion process, especially for ACR steel tubing, despite its similarities to copper tube expansion [11,12].

Studies by Almeida et al. and Alves et al. have evaluated tube thickness reduction and the impact of mandrel shapes on expansion forming, incorporating numerical models and experimental tests for validation [13,14]. Tube expansion has also been applied to plastic pipes, as investigated by Alves in PVC tube expansion with mandrels [15]. An analytical model predicting the push force required for tube expansion, based on Hoop stress reaching yield, uses a rigid–linear strain hardening material behavior [16]. Bouzid et al. proposed an analytical model for estimating residual stresses in hydraulically expanded tube-to-tubesheet joints, highlighting the impact of reverse yielding on residual contact stresses [17,18]. The kinematic versus isotropic material behaviors of tube expansion of finned tubes were explored in theoretical studies, addressing stresses in the transition zone but not the high stresses in the contact zone where the expander meets the tube [19,20]. The significance of stresses in curved components in industrial piping systems is also emphasized in the article by Fonseca et al. [21], who present a semi-analytic method to calculate flexibility factors in curved pipes with end restraints.

Previous analytical studies primarily focused on idealized material properties of thin-walled tubes, often neglecting the complexities of nonlinear plastic deformation, applying a maximum shear criterion and analyzing only the plastic zone. This paper outlines a methodology for estimating the stresses and strains during expansion in the different tube zones, supported by numerical finite element modeling and experimental data on copper and stainless steel 316 tubes expanded with an ogive bullet. This research builds on foundational work by extending an analytical model to incorporate nonlinear plastic deformation of relevant materials to heat exchangers and gas coolers while applying the von Mises criteria. This enhanced approach explores the impacts of mandrel shapes and tube thickness on mechanical behaviors in high-stress industrial applications. This approach, together with consistent findings in the analytical, numerical, and experimental methods, advances the understanding of tube expansion processes and address significant research gaps.

## 2. Analytical Model

**Figure 1:**
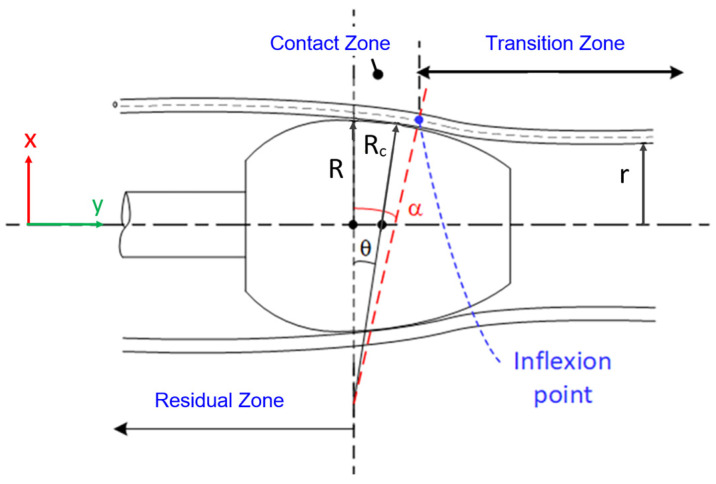
Different zones in an expanded tube.

The expanded tube region is categorized into three zones: the transition zone, the contact zone, and the residual one, as depicted in Figure 1. Critical to this model are the initial contact point of the tube with the expanding bullet and the radial expansion force, both essential for estimating stresses and driving forces. A self-adaptive method assesses the stress distribution within the transition zone, while the Prandtl–Reuss flow rule of plasticity evaluates stresses in the contact zone.

The boundaries delineating the three expansion zones are characterized by distinct demarcations. The transition zone encompasses the unexpanded portion of the tube up to the point where the mandrel makes initial contact, encompassing the elastic, partially plastic, and fully plastic regions. The boundary between the contact and residual stress zones is defined by the onset of separation between the tube and the mandrel.

Expanding mandrel configurations typically manifest as spherical or ogival shapes and are commonly delineated within a Cartesian coordinate framework. In Figure 1, the boundary separating the transition and contact zones is depicted as tangential to the mandrel surface. At this juncture between the contact and transition zones, the tube exhibits both a rotational angle ($\theta_{c}$) and radial displacement ($y_{c}$), as as illustrated in Figure 1. These parameters are interconnected by Equation (1) that considers a curved mandrel shape with a radius of curvature $R_{c}$. r is the inner radius of the tube and R is the outer radius of the mandrel.

(1) $$y_{c}=R_{c}\left(\cos\theta_{c}-1\right)-r+R$$

The radial displacement and rotation of the tube can be obtained using beam on elastic foundation theory for the transition zone.

### 2.1. Transition Zone Analysis

**Figure 2:**
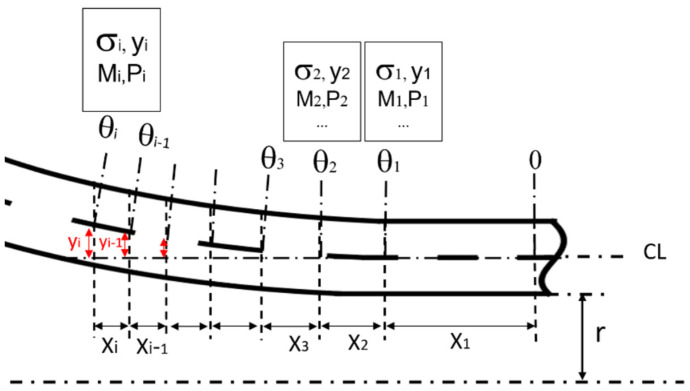
Deformations in the tube transition zone.

In Figure 1, the transition zone is delineated into two primary regions: the elastic and plastic regions. The calculation approach for both regions within the transition zone remains consistent. According to findings from a prior investigation [18], it was discerned that the equivalent stress in the tube either reaches or exceeds the yield point, even with minimal radial expansion. Consequently, the tube undergoes strain hardening to a certain degree before coming into contact with the mandrel. Due to the relatively diminutive size of this zone, its behavior can be approximated effectively using the secant modulus during the loading phase. Moreover, the self-adaptive method is employed for stress estimation. Consequently, the transition zone is subdivided into multiple sections of unequal lengths, each characterized by a distinct radial displacement and rotation angle, as depicted in Figure 2. While the rotation angle $\theta_{c}$ of the tube at the point of contact aligns tangentially with the mandrel surface, it remains initially unknown for an ogive bullet. Therefore, the rotation of each section is iteratively incremented by

(2) $$\theta_{i}=\theta_{i-1}+∆\theta$$

**Figure 3:**
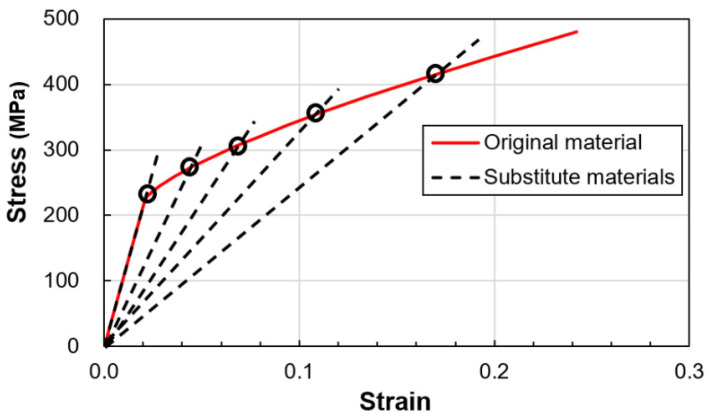
Simplified material behavior.

The stress $\sigma_{i}$ and the corresponding secant modulus $E_{i}$ for each section are derived from the stress–strain curve. Achieving convergence for rotation and displacement necessitates an iterative procedure. This iterative approach, known as the self-adaptive process, is instrumental in estimating stresses accurately. The von Mises effective stress is employed alongside a power-law strain hardening model. Material data conform to ASTM standard E646, and the true stress–strain curve is fitted with a nonlinear hardening Ludwick equation to ensure comprehensive representation.

(3) $$\begin{array}{cc} \sigma_{e}=E\varepsilon_{e} & for\left(\varepsilon_{e}\leq \varepsilon_{y}\right)\\ \sigma_{e}=\sigma_{y}+K{\left(\varepsilon_{e}-\varepsilon_{y}\right)}^{n_{o}} & for\left(\varepsilon_{e}>\varepsilon_{y}\right) \end{array}$$

where $\sigma_{y}$ and $\varepsilon_{y}$ are the yield stress and strain and K and $n_{o}$ are the stain hardening constant and exponent, respectively, of the Ludwick power law. The stress condition for each section of the tube is that the equivalent stress is equal to the current stress $\sigma_{i}$. The secant modulus of the transition zone shown in Figure 3 is defined by:

(4) $$\begin{array}{cc} E_{i}=E & for\left(\varepsilon_{e}\leq \varepsilon_{y}\right)\\ E_{i}=\frac{\sigma_{i}}{\sqrt[{n_{o}}]{\frac{\sigma_{i}-S_{y}}{K}}+\varepsilon_{y}} & for\left(\varepsilon_{e}>\varepsilon_{y}\right) \end{array}$$

The theory of a beam on an elastic foundation applied to a cylinder is used to obtain the following four parameters as a function of the length, x: radial displacement $y_{i}$; rotation $\theta_{i}$; moment $M_{i}$; and shear force $P_{i}$ [3]:

(5) $$y_{i}=\frac{2P_{i}\beta_{i}}{k_{i}}D_{\beta x}+\frac{{2M}_{i}\beta_{i}^{2}}{k_{i}}C_{\beta x}$$

(6) $$\theta_{i}=\frac{2P_{i}\beta_{i}^{2}}{k_{i}}A_{\beta x}+\frac{4M_{i}\beta_{i}^{3}}{k_{i}}D_{\beta x}$$

(7) $$M_{i}=\frac{P_{i}}{\beta_{i}}B_{\beta x}+M_{i}A_{\beta x}$$

(8) $$P_{i}=P_{i}C_{\beta x}+2\beta_{i}M_{i}A_{\beta x}$$

where

(9) $$\beta =\frac{\sqrt[{4}]{3\left(1-\nu^{2}\right)}}{\sqrt{rt}},k_{i}=\frac{E_{i}t}{r^{2}}$$

The influence coefficients are given by

(10) $$\begin{array}{c} A_{\beta x}=e^{-\beta x}\left(\cos\beta x+\sin\beta x\right)\\ B_{\beta x}=e^{-\beta x}\sin\beta x\\ C_{\beta x}=e^{-\beta x}\left(\cos\beta x-\sin\beta x\right)\\ D_{\beta x}=e^{-\beta x}\cos\beta x \end{array}$$

The longitudinal and tangential stresses $\sigma_{li}$ and $\sigma_{\theta i}$ are given as a function of the edge loads $P_{i}$ and $M_{i}$ for each section:

(11) $$\sigma_{li}=\pm \frac{6M_{i}}{t^{2}}$$

(12) $$\sigma_{\theta i}=\frac{6{\nu M}_{i}}{t^{2}}+\frac{E_{i}y}{r}$$

(13) $$\sigma_{i}=\sqrt{{\sigma_{li}}^{2}+{\sigma_{\theta i}}^{2}-\sigma_{li}\sigma_{\theta i}}$$

For thin tubes, simplifying the analysis involves averaging stresses across the thickness. Employing the theory of a beam on an elastic foundation, the process occurs in two steps for each section: first, calculating stresses in section i considering its end conditions and then, using information from the preceding step and conditions of section i − 1 for length $x_{i-1}$, determining the size of the current section $x_{i}$, depicted in Figure 2. This method offers an estimate of stress distribution in the transition zone. Its advantage lies in integrating material hardening and stress theory previously applicable only under elastic conditions, thereby offering a simplified solution to evaluate the stresses in the elastic-plastic transition zone.

### 2.2. Contact Zone Analysis

**Figure 4:**
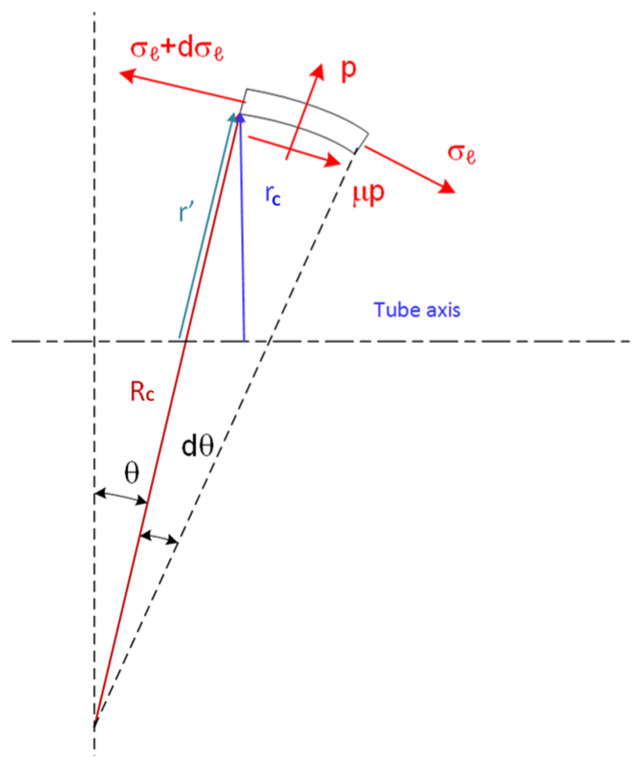
Simplified material behavior.

The analysis of the tube’s plastic contact zone entails solving a system of six equations with six unknowns. These equations stem from considerations of axial and radial equilibrium, the plasticity Prandtl–Reuss flow rule, the volume conservation equation of plasticity, and the Von Mises equivalent stress–strain relationship. In this analysis, shear effects are disregarded, rendering the normal radial, tangential, and longitudinal stresses $\sigma_{r}{,\;\sigma }_{\theta },\;\sigma_{l}$ principal stresses. Using membrane theory,

(14) $$\frac{\sigma_{l}}{R_{c}}+\frac{\sigma_{\theta }}{r^{\prime }}=\frac{p}{t}$$

where p and t are the pressure and tube thickness and $r^{\prime }$ is a radius given from the geometry shown in Figure 4.

(15) $$r^{\prime }=R_{C}-\frac{R_{c}-R}{cos\theta }$$

The free-body diagram of the plastic region shown in Figure 4 gives the equilibrium equation in the x-direction including the friction component as

(16) $$\frac{d\sigma_{l}}{d\theta }+\sigma_{l}sin\theta \frac{R_{C}+r^{\prime }}{R_{C}\left(cos\theta -1\right)+R}=p\frac{R_{C}}{t}(\mu +tan\theta )$$

The conservation of the volume due to the plastic behavior gives:

(17) $$\varepsilon_{\theta }+\varepsilon_{l}+\varepsilon_{r}=0$$

The tangential strain is given by

(18) $$\varepsilon_{\theta }=\log\frac{r_{c}}{r}=log\frac{r^{\prime }}{rcos\theta }$$

The application of the Prandtl–Reuss flow rule of the plastic deformation region is

(19) $$\frac{\varepsilon_{\theta }}{\sigma_{\theta }-\sigma_{m}}=\frac{\varepsilon_{l}}{\sigma_{l}-\sigma_{m}}=\frac{\varepsilon_{r}}{\sigma_{r}-\sigma_{m}}$$

The mean stress is given by

(20) $$\sigma_{m}=\frac{1}{3}\left(\sigma_{l}+\sigma_{\theta }+\sigma_{r}\right)$$

The radial stress is assumed to be the average normal stress between the inside and outside surfaces:

(21) $$\sigma_{r}=-\frac{p}{2}$$

Using the Von Misses effective stress and strain,

(22) $$\sigma_{e}=\sqrt{\frac{1}{2}\left[{\left(\sigma_{\theta }-\sigma_{l}\right)}^{2}+{\left(\sigma_{l}-\sigma_{r}\right)}^{2}+{\left(\sigma_{r}-\sigma_{\theta }\right)}^{2}\right]}$$

The equivalent strain is given by

(23) $$\varepsilon_{e}=\sqrt{\frac{2}{3}\left[{\left(\varepsilon_{\theta }-\varepsilon_{l}\right)}^{2}+{\left(\varepsilon_{l}-\varepsilon_{r}\right)}^{2}+{\left(\varepsilon_{r}-\varepsilon_{\theta }\right)}^{2}\right]}$$

By substituting Equations (22) and (23) into Equation (3), and Equation (21) into Equations (14) and (16), a system of six non-linear equations is derived with six unknowns: $\varepsilon_{r},{\;\varepsilon }_{\theta },\;{\;\varepsilon }_{l},{\;\sigma }_{r},{\;\sigma }_{\theta },\;$and $\sigma_{l}$. Utilizing Equations (14) and (16)–(19), this system is solved using the Matlab 2019b solver with appropriate initial conditions. By iteratively adjusting the radius r and solving the system, the stress distribution within the contact zone is determined. Furthermore, the axial pull or push force is computed by integrating the friction force over the plastic contact area expanded with the mandrel:

(24) $$F=2\pi R_{c}\;\int_{0}^{\theta_{c}}p(R_{c}\left(cos\theta -1\right)+R)\left(\mu cos\theta +sin\theta \right)d\theta$$

## 3. Numerical Model

The finite element method (FEM) serves as the validation framework for our analytical approach, focusing on two types of tubes with a 3/8 inch outside diameter and a 0.035 inch thickness: copper C122 and stainless steel SS316L. Copper C122 is widely utilized in manufacturing low-pressure gas cooler exchangers due to its excellent thermal conductivity and corrosion resistance. Conversely, stainless steel SS316L is being explored for high-pressure applications, particularly in exchangers employing CO_2_ as a less polluting refrigerant, owing to its superior strength and corrosion resistance.

**Figure 5:**
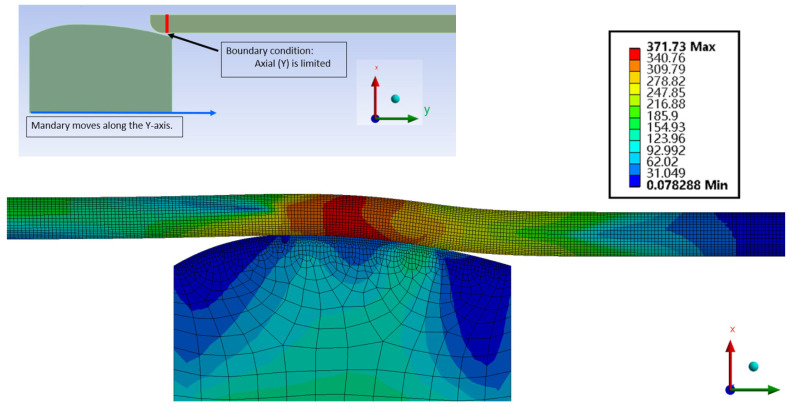
FE model of tube and expanding bullet: equivalent stresses (MPa).

The model is mainly composed of QuadShell8 elements, with a total of 9468 elements. The QuadShell8 element offers several advantages, including an increased accuracy over Quad4 or Triangular elements. A refined finite element mesh, detailed in Figure 5, models the tubes and the expanding bullet based on dimensions and materials specified in Table 1. A mesh refinement criterion of 1% based on the equivalent stress of the most highly stress node was used. The stress–strain behavior, adhering to Ludwick’s material plasticity model, is characterized by the hardening constants also listed in Table 1 For this simulation, we employ a multilinear kinematic strain hardening model to accurately capture the material’s response under varying load conditions. The stress–strain data are obtained by interpolation using Formula (3) mentioned above. A friction coefficient of 0.29 is assumed during the tube expansion process to account for the interaction between the tube and mandrel surfaces.

The simulation constraints include preventing axial movement at one end of the tube—where expansion initiates—while allowing for radial expansion and axial retraction at the opposite end. This setup mimics the real-world application where one end of the tube is fixed to a point, such as a tubesheet, while the other end can expand or contract freely. The expanding bullet’s motion is modeled to traverse from one end of the tube to the other at a speed of 2 mm/s, a parameter recommended by local industry experts and corroborated by experimental data.

## 4. Experimental Testing

**Figure 6:**
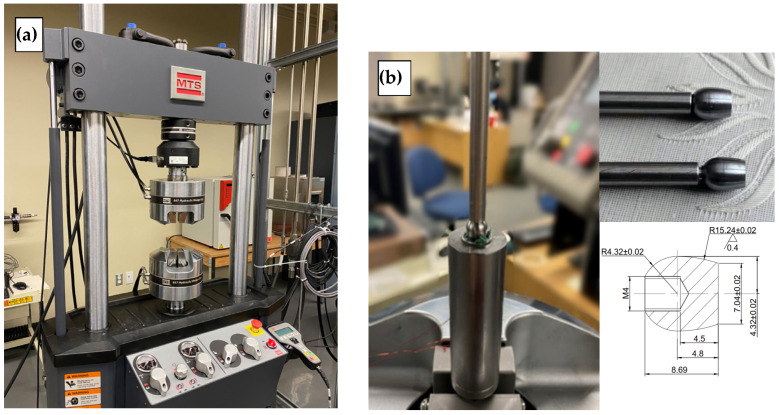
Experimental set-up: (**a**) Hydraulic tensile compression machine. (**b**) Tube expansion set and mandrel details (unit: mm).

To validate the analytical and numerical models, experimental tests were conducted on 3/8 in. SS316 and copper C122 tubes using an ogive bullet on a specialized test workbench equipped with a hydraulic tensile/compression testing of an MTS machine, as shown in Figure 6a. The setup involves fixing the mandrel with its rod to the upper grip of the test machine, while the tube guide, specifically designed for 3/8 in. tubes and depicted in Figure 6b, is attached to the lower grip. This arrangement ensures the tube is adequately supported during the expansion process, whether being pushed or pulled, with the guide restraining the tube at the appropriate end.

**Figure 7:**
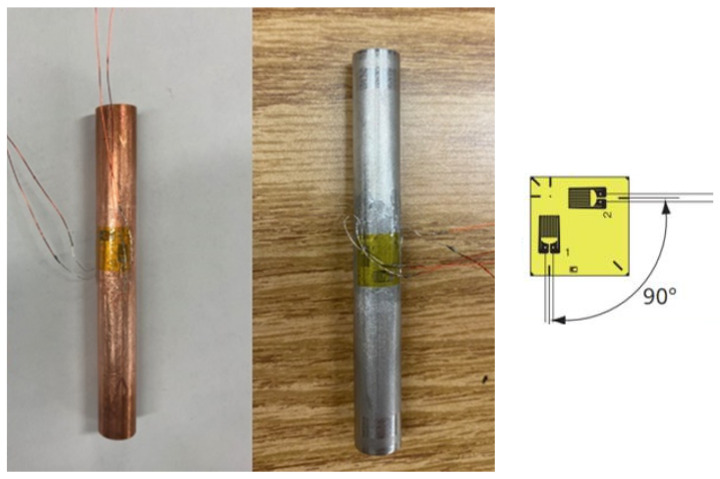
Tee strain-gauged copper C122 and SS316L tubes.

**Figure 8:**
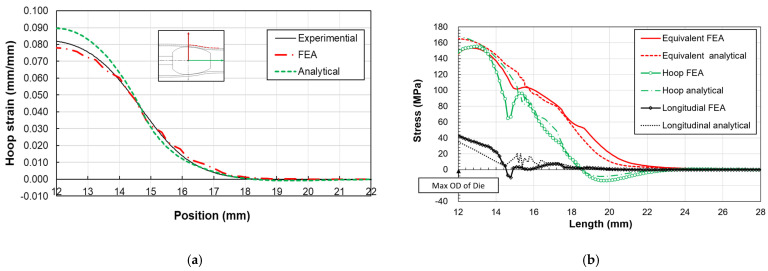
Comparison of copper C122 tube: (**a**) hoop strain outside surface, (**b**) stress distribution along axial direction.

Instrumented with strain gauges, the tubes are placed within the guide for expansion, as illustrated in Figure 7. A tee rosette strain gauge, with a 2 mm width capable of measuring up to 15% strain, is bonded to the tube’s external surface to monitor axial and Hoop strains during the expansion shown in Figure 8. These strains are continuously observed in a quarter bridge configuration, allowing for precise tracking as the expanding bullet progresses through the tube. The experimental setup enables the simultaneous recording of the driving force, axial and Hoop strains, and time at regular intervals, providing a comprehensive dataset for validating the theoretical and numerical analyses.

The MTS machine utilized in this experiment features a data acquisition system capable of recording and monitoring relevant data in real time. This system is equipped to record data at frequencies of 1, 10, or 100 times per second. Given the die’s slow movement speed of 2 mm/s in our experiment, a recording frequency of 10 times per second is deemed adequate for fulfilling the validation requirements. Before the experiment, the contact between the mandrel and the inner wall of the pipe was lubricated using oil. During the expansion process, the driving force, the axial and hoop strains and axial distance are monitored through the data acquisition system.

## 5. Results and Discussion

This study introduces an analytical method designed to assess stress and strain distributions within the transition and contact zones of expanded tubes. By juxtaposing the results derived from this analytical approach with those gleaned from numerical finite element (FE) models and experimental investigations, the study aims to validate the efficacy of the proposed method.

### 5.1. Copper C122 Tube Expansion

The expansion of a 3/8 in, gauge 20, copper C122 tube using an ogive is examined. Despite the high radius-to-thickness ratio of 4.35, which typically renders thin shell theory less applicable, the analytical method still provides a reasonably accurate estimation of stress and strain within the contact and transition zones. The hoop strain results, as illustrated in Figure 8, reveal a good alignment between analytical and FEM predictions compared to experimental data. The maximal hoop strain, identified at the ogive’s largest radius, is measured as approximately 8% in experimental measurements. A notable discrepancy, about 10%, between analytical and experimental values at this juncture is attributed to the analytical model’s reliance on an average radius, whereas experimental measurements target the tube’s outer surface. The stress distribution along the tube length, encompassing both transition and contact zones, as depicted in Figure 8, demonstrates that analytical and numerical stress distributions, averaged across the tube thickness, remain comparable. While congruence is observed in the longitudinal, hoop, and equivalent stresses between both methods, disparities emerge at the boundary of the contact and transition zones. These differences likely stem from unaccounted bending stresses in the analytical model, particularly at points where curvature changes occur between zones and at the maximum ogive radius, where tube bending and stress release happen.

### 5.2. Stainless Steel 316L Tube Expansion

**Figure 9:**
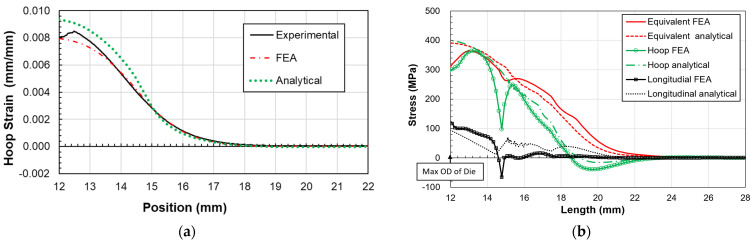
Comparison of stainless-steel 316L tubes: (**a**) hoop strain outside surface, (**b**) stress distribution along axial direction.

Similarly, a stainless steel 316L tube undergoes expansion via the same ogive for comparative analysis. The hoop strain, showcased in Figure 9, mirrors the pattern observed in the copper tube, with the highest strain occurring at the ogive’s maximum diameter. The experimental data indicate a maximal strain of about 8.6%, while the analytical model predicts 9.3%. Despite some deviations, notably an 8% difference at the peak ogive diameter, both analytical and FE results largely concur with experimental findings. A notable drop in experimental strain at this peak suggests potential adhesion loss between the tube and the strain gauge, a problem that might be mitigated by employing adhesives suitable for high-strain scenarios.

The stress distributions within the stainless steel 316L tube’s expanded zones, outlined in Figure 9, show good agreement between analytical and FE models in the transition zone. However, less correlation is observed at the interface between the transition and contact zones, and at the maximum outer diameter (OD) position, attributed to bending moments not accounted for in the analytical model.

**Figure 10:**
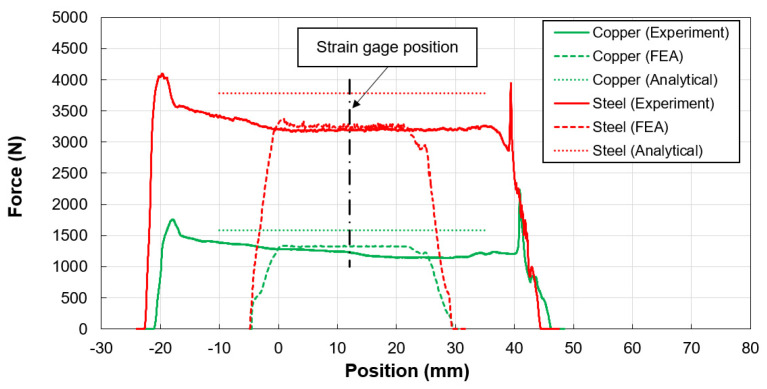
Expanding mandrel force measurements and comparison.

The expander driving force, evaluated through contact zone pressure distributions and presented in Figure 10, exhibits initial spikes attributed to end effects, such as sharp tube edges and inadequate lubrication, before stabilizing at lower values once the mandrel is fully inserted into the tube. Although FEM results indicate similar stabilization, the forces estimated by the analytical model slightly exceed those measured and averaged by FEM, likely due to overestimations in radial stress or contact pressure and the model’s inability to simulate bending effects accurately.

## 6. Conclusions

This study has introduced an analytical model designed to accurately estimate stresses and strains within the transition and contact zones of tubes undergoing tube expansion. The robust alignment of results obtained from analytical, numerical, and experimental evaluations underscores the reliability of the developed method. Specifically, the model predicts strains with a deviation of less than 10% and delivers stress estimates that closely mirror those observed, especially in regions where curvature changes are pronounced, such as the interfaces between transition and contact zones and between contact and residual stress zones. While there are some discrepancies in the contact zone between numerical and experimental results, the model gives a relatively good estimate of the stresses and strains. Nevertheless, the differences are likely due to the following factors. Firstly, the thickness of the strain gauge adhesive and the bonding strength can affect the curvature at maximum deformation, influencing the measurement results. Secondly, the two-dimensional simulation does not fully capture the three-dimensional deformation of the tube under high expansion and particularly the changes in the thickness. These issues, while noteworthy, do not significantly detract from the overall effectiveness and applicability of the model.

The application of beam-on-elastic-foundation theory, supplemented by the self-adaptation method, has proven effective in modeling the low-plastic regions, exemplified by the transition zone. This approach has demonstrated its versatility across different materials, including copper and stainless steel, and has shown to be applicable even to thin-walled tubes, despite the inherent challenges posed by a tube radius-to-thickness ratio of 4.35 in the studied cases.

The stress levels within the contact zone, identified as reaching maximal values, underscore this area as critically important for monitoring to prevent potential necking during the expansion process. The findings affirm that the proposed analytical model is a valuable tool for predicting stress and strain distributions in tube expansion scenarios, offering significant insights for ensuring the structural integrity of expanded tubes in practical applications.

## Figures and Tables

**Table 1:** Dimensions and material properties [22,23].

Property	Tubes 3/8 in ×0.035 in		Mandrel
	Copper C122	SS 316L	Steel M4
R (mm)	-	-	4.32
R_c_ (mm)	-	-	15
r (mm)	3.8735	3.8735	-
t (mm)	0.889	0.889	-
E (GPa)	112	206	214
ν	0.34	0.25	0.29
S_y_ (MPa)	69	175	800
B (MPa)	455	800	-
n	0.68	0.575	-
μ (friction)	0.28	0.29	

## Data Availability

The data presented in this study are available on request from the corresponding author due to privacy.
